# FAP-targeted PET imaging in gastrointestinal malignancies: a comprehensive review

**DOI:** 10.1186/s40644-023-00598-z

**Published:** 2023-08-22

**Authors:** Ayça Arçay Öztürk, Patrick Flamen

**Affiliations:** Department of Nuclear Medicine, Institut Jules Bordet, Université Libre de Bruxelles, Brussels, Belgium

**Keywords:** Fibroblast activation protein, Cancer-associated fibroblasts, FAPI, PET, Gastrointestinal oncology, FDG PET/CT, FAPI PET/CT, Gastric cancer, Colorectal cancer

## Abstract

F18-fluorodeoxyglucose positron emission tomography/computed tomography (FDG PET/CT) plays a crucial role in tumour diagnosis, staging, and therapy response evaluation of various cancer types and has been a standard imaging modality used in clinical oncology practice for many years. However, it has certain limitations in evaluating some particular gastrointestinal cancer types due to low FDG-avidity or interphering physiological background activity. Fibroblast activation protein (FAP), a protein of the tumour microenvironment, is overexpressed in a wide range of cancers which makes it an attractive target for both tumour imaging and therapy. Recently, FAP-targeted radiopharmaceuticals are widely used in clinical research and achieved great results in tumour imaging. Considering the limitations of FDG PET/CT and the lack of physiological FAP-targeted tracer uptake in liver and intestinal loops, gastrointestinal cancers are among the most promising indications of FAP-targeted imaging. Herein, we present a comprehensive review of FAP-targeted imaging in gastrointestinal cancers in order to clarify the current and potential future role of this class of molecules in gastrointestinal oncology.

**Supplementary Information:**

The online version contains supplementary material available at 10.1186/s40644-023-00598-z.

## Background

Cancer morbidity and mortality are rapidly increasing in recent years, approximately 19.3 million new cancer cases and 10 million cancer deaths occurred worldwide in 2020 [1]. Cancers compose of malignant cells and surrounding stroma, which consists of non-malignant cells and accounts for a large part of the tumour mass. However, diagnostic and therapeutic approaches have predominantly targeted only malignant cells. Very recently, there has been increased attention in cancer research being paid to the tumour stroma, the so-called tumour microenvironment.

CAFs (cancer-associated fibroblasts) are one of the major cellular components of the tumour stroma. These cells are fibroblasts activated by tumour-promoting inflammation and tumour fibrosis in cancer tissue [2]. Developing a molecule targeting CAF achieved wide attention since they have a critical role in tumour growth, progression, and migration. Additionally, in contrast to normal fibroblasts, CAFs express particular proteins that can be used as tumour-specific molecular target. FAP (fibroblast activation protein) is one of them, which is a type II transmembrane serine protease and highly expressed in CAFs. FAP overexpression has been demonstrated in most of the epithelial cancers, especially in tumours with a high degree of desmoplasia [3]. The extensive expression of FAP makes it an attractive target for both imaging and therapy of a broad spectrum of tumours.

At present, PET/CT is a commonly used imaging modality in clinical oncology practice and a glucose analog, fluorodeoxyglucose (FDG) labeled with Fluorine-18 (F18) is the dominant tracer in detecting malignancy on PET/CT. However, FDG PET/CT has certain drawbacks in particular indications such as gastric mucus adenocarcinoma, highly differentiated hepatocellular carcinoma, and peritoneal metastasis.

Several FAP-targeting radiopharmaceuticals for imaging and therapeutical applications have already been developed [4–9]; the most commonly used radiotracers are small molecule fibroblast activation protein inhibitors (FAPI) labeled with Gallium-68 or Fluorine-18 [10]. The low tracer uptake in normal healthy tissues and the high uptake in malignant tissues result in high contrast between the tumour and background, allowing for good tumour delineation. Moreover, FAPI PET offers advantages over FDG PET regarding patient preparation, as it does not necessitate fasting before the scan and early imaging (e.g. 10 min post-injection) is feasible [11, 12]. FAP-targeted imaging has so far shown to have impressive results and up-and-coming potential in a wide range of cancers [13, 14].

According to the findings of previous studies, gastrointestinal cancers are among the most promising indications of FAP-targeted imaging [13–16]. Therefore, this review aims to provide a general impression of how FAP-targeted imaging can affect the diagnosis and treatment management of gastrointestinal cancers as well as to give an idea about future research directions on this topic.

## FAP-targeted radiopharmaceuticals

The first clinical FAP-targeting was reported by Welt et al. in 1994 by performing planar and SPECT imaging with a I131-labeled monoclonal murine antibody mAb F19 in 17 patients. They declared that the highly selective expression pattern allows imaging of colorectal lesions as small as 1 cm in diameter on I131-mAbF19 scans [17]. After that, several radiolabeled antibodies and peptides targeting FAP has been developed [18, 19]. However, a comprehensive application in nuclear medicine couldn’t be achieved due to the long circulation and slow clearance caused by their high molecular mass.

This led to the introduction of small molecules. In 2014, the group of van der Veken at the University of Antwerp developed UAMC-1110 which is a highly potent FAP inhibitor. This small molecule FAP inhibitor demonstrated low nanomolar FAP affinity and high selectivity toward related enzymes prolyl oligopeptidase and dipeptidyl-peptidases [20, 21]. FAPI (FAP inhibitor) precursors and various FAPI tracers were designed based on this motif by the Haberkorn group at the University of Heidelberg [4, 5]. They first developed two radiotracers, a radioiodine-labeled FAPI, FAPI01, and, a precursor for the chelation of radiometals, FAPI02 [4]. The I125-FAPI01 was no longer included in preclinical studies because of its time-dependent efflux and enzymatic deiodination. FAPI02 has high tumour specificity but declining uptake over time. To overcome this problem, the same group developed a series of piperazine-based FAP inhibitors labeled with the positron emitter Ga68. Of 15 synthesized FAPIs, FAPI04 was identified as the most promising tracer for clinical application [5]. They reported the effective tumour uptake after 24 h as 100% higher for FAPI04 than for FAPI02. In order to improve the potential therapeutic efficacy through higher dose delivery, 15 more FAPI variants were designed to further increase tumour uptake and retention of these tracers by Heidelberg group [6]. Of these 15 FAPIs the overall improved tumour–to–normal-tissue ratios were achieved with FAPI21 and FAPI46. FAPI46 proved to be more favorable as a theranostic agent due to the increased uptake of FAPI21 in normal organs such as the thyroid, oral mucosa, and salivary glands [6].

Besides them, novel FAP-targeting radiotracers were developed using bifunctional DOTA and DATA5m chelators coupled by squaramide as a linker moiety [7]. Ga68-DOTA.SA.FAPi is reported as a promising alternative among the FAPI molecules and good performance has been demonstrated as compared to F18-FDG in the diagnosis of various cancers [22]. However, the first theranostic approach of Ga68-DOTA.SA.FAPi PET/CT and Lu177-DOTA.SA.FAPi revealed the early washout of the radiotracer and this was the major disadvantage of the molecule [23]. To overcome this drawback, Moon et al. modified the structure and introduced dimeric systems for prolonged tumour retention. Using the SA.FAPi monomer as the base, they developed two homodimeric structures such as DOTA(SA.FAPi)2 and DOTAGA.(SA.FAPi)2 [24]. Lu177-DOTAGA.(SA.FAPi)2 had a significantly longer median whole-body effective half-life compared to that of [177Lu]Lu-DOTA.SA.FAPi (46.2 h vs. 23.1 h; *p* = 0.0167), subsequent clinical dosimetry study demonstrated significantly higher tumour absorbed doses with Lu177-DOTAGA.(SA.FAPi)2 compared to Lu177-DOTA.SA.FAPi [25].

Ga68-FAPIs have already been proposed as promising PET tracers. However, PET imaging with Ga68-labeled radiotracers has a drawback regarding radionuclide supply. Due to the limited batch production capacity of Ge68/Ga68 generators, the potential demand of high patient throughput centers may not be met and the delivery to remote centers is challenging because of the relatively short half-life (68 min) of Ga68. Additionally, F18 has lower positron energy than Ga68, which leads to a shorter positron range and eventually higher spatial resolution. Therefore, FAPI molecules radiolabeled with F18 have been developed, namely, F18-FAPI-74, F18-FGlc-FAPI and F18-FAPI-42. FAPI-74 ligand has NOTA as the chelator, this enables it to be labeled with both AlF-F18 and Ga68 which leads to flexible routine use. FAPI-74 was developed as a solely diagnostic ligand, accepting slightly shorter tumour retention than the previous theranostic agents such as FAPI04 and FAPI46 [8]. The glycoconjugate F18-Glc-FAPI was shown to have higher plasma protein binding and lipophilicity than Ga68-FAPI04 which results in lower tumour-to-background ratios due to the slower blood clearance [26]. Furthermore, because F18-Glc-FAPI is excreted through the kidneys as well as the hepatobiliary pathway, nonspecific uptake of F18-FGlc-FAPI in the liver and intestine may pose a problem in detecting abdominal FAP-positive lesions. Despite these disadvantages, high uptake of F18-Glc-FAPI in bone structures which was observed in the same preclinical study may be beneficial in diseases such as rheumatoid arthritis [26].

Besides FAPI homodimers (e.g. DOTA(SA.FAPi)2, DOTAGA.(SA.FAPi)2); heterodimers such as FAPI-RGD (arginine-glycine-aspartate) targeting both FAP and αvβ3 were also developed to enhance tumour uptake and retention [27].

Several solutions have been implemented in the literature in order to increase the tumour retention of the tracer. One of them was the previously mentioned dimerization of FAPI (e.g. DOTA(SA.FAPi)2, DOTAGA.(SA.FAPi)2) [24, 25]. Another solution was done to increase the bloodstream circulation by albumin binding (e.g. Evan’s Blue conjugates [28]). Furthermore, different classes of molecules such as cyclic peptides like FAP-2286 have been studied on [9, 29]. Preclinical and clinical studies with those compounds are still ongoing.

## FAP-targeted imaging in gastrointestinal oncology

### Esophageal cancer

FDG PET/CT is reliable in remote lymph node and distant metastases detection in esophageal cancer, whereas it is less reliable for locoregional lymph node detection [30, 31]. Another drawback for the interpretation of FDG PET in esophageal cancer is the false positive tracer uptake in active inflammation (e.g. reflux oesophagitis; post radiotherapy).

FAP immunohistochemistry (IHC) scoring demonstrated strong FAP expression in 50–100% of esophageal cancer cases, which was declared to be one of the highest among the fourteen investigated cancer types [32]. Accordingly, one of the highest average SUVmax (> 12) was found in esophageal cancer on Ga^68^-FAPI PET [33]. Notably, the existing FAPI PET literature primarily focused on esophageal squamous cell carcinoma, the predominant subtype in Central and Eastern Asia.

In terms of primary tumour uptake, Zhao et al. reported higher standardized uptake values (SUVs) in the lower esophageal tumours on FAPI PET than on FDG PET. However, no statistically significant difference was detected in the cervical and upper esophageal tumours [34]. In another study with 35 esophageal cancer patients, Liu et al. also declared the superiority of primary tumour SUVs and detection sensitivity on FAPI PET compared to FDG PET [35] (Table 1 and Supplementary Table 1- Additional file 1).

**Table 1:**
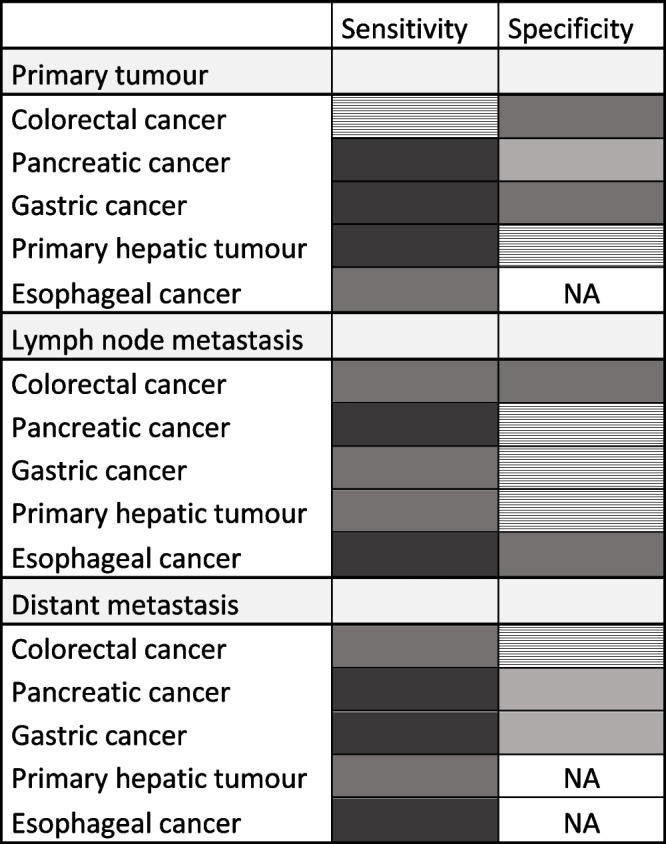
Sensitivity and specificity comparison of FAPI PET and FDG PET in gastrointestinal cancer lesions

Table 1 shows the sensitivity and specificity comparison between FAPI PET and FDG PET imaging in each considered cancer type including the detection of primary tumour and metastatic lesions. This conclusion was derived from the published studies of FAPI vs. FDG comparison which were referred throughout the text. The darkest gray colour indicates “FAPI is superior to FDG”. The lighter gray colour indicates “FAPI surpasses FDG although there are non-significant or controversial findings”. The lighest gray colour indicates “FAPI is inferior to FDG including non-significant findings in some studies”. The striped box indicates “FAPI is comparable to FDG”. “NA” means that enough data is not available

Moreover, Zhao et al. reported that SUVs of metastatic lymph nodes were significantly higher on FAPI compared to FDG PET. Also the detectability of lymph node metastasis was higher on FAPI PET although not statistically significant [34]. The good diagnostic performance of FAPI PET in detecting lymph node metastases in esophageal cancer was also shown in 44 patients cohort with a sensitivity of 81.5% and a specificity of 99.3% [36].

Liu et al. confirmed these findings, both SUVs and detectability of lymph node metastasis were reported as significantly higher on FAPI PET than that of FDG PET. Similar results were also declared for bone and visceral metastatic lesions in the same study [35] (Supplementary Table 1- Additional file 1).

Considering the higher primary tumour uptake on FAPI PET, one of the major points in the investigation of esophageal cancer was directed to the tumour delineation for radiotherapy. Although the available data is not robust due to limited number of patients, Ristau et al. reported that FAPI PET improved target tumour delineation in six out of seven patients and alteration of radiotherapy planning in nearly all patients, mostly due to change in gross tumour volume (GTV) compared to standard CT imaging [37]. Zhao et al., in their study including 21 esophageal cancer patients, concluded that FAPI PET showed favorable image contrast in esophageal cancer and might be used as a complementary imaging tool for target delineation [34].

In addition, the same study group, in another study including 34 patients, investigated the prognostic value of semiquantitative parameters derived from FAPI PET for esophageal squamous cell carcinoma treated with definitive chemoradiotherapy. Among SUVmax, gross tumour volume (GTV), and total lesion-FAPI (TL-FAPI), GTV was found to be an independent prognostic factor for both progression-free survival and overall survival. This finding which is based on the high tumour-to-background ratio (TBR) obtained with FAPI PET provides another rationale for the application of FAPI PET for delineation of target volumes for radiotherapy [38]. There is also another prospective study similar to the aforementioned study, which examined the predictive value of quantitative parameters derived from FAPI PET on short-term outcomes in locally advanced esophageal squamous cell carcinoma patients receiving concurrent chemoradiotherapy [39]. Among SUVmax, SUVmean, SUVpeak, metabolic tumour volume (MTV), total lesion FAP expression (TLF), tumour-to-blood background ratio (TBRblood), and tumour-to-muscle background ratio (TBRmuscle), TBRblood was reported to be an independent prognostic factor for short-term outcome [39].

Hence, FAPI PET has promising potential regarding better tumour delineation of the esophageal primary tumour for radiotherapy planning and also superiority in terms of primary and metastatic lesions’ uptake and detectability. In addition, it is shown that quantitative parameters derived from FAPI PET can have prognostic value in the esophageal cancer patient group.Further prospective well controlled studies are needed in this topic. Data about use of FAPI PET in oesophageal adenocarcinoma patients, for therapy monitoring and recurrence detection are largely missing (Fig. 3).

### Gastric cancer

FDG PET/CT has been shown to contribute in nodal staging and metastatic status of the disease in gastric cancer. However, primary gastric cancer has been demonstrated to have a low detection rate by FDG PET (~ 55%), particularly in early stages, signet ring cell and mucinous carcinoma histologic types. Moreover, variable physiological FDG uptake in gastric wall can mask primary tumours [40].

Gastric cancer exhibited strong FAP expression in 50% of the cases [32]. Gastric cancer uptake on FAPI PET was comparatively lower than the other gastrointestinal tumors and ranked among the lowest (average SUVmax < 6) in 28 cancer types [33], however, the high tumour-to-background ratio on FAPI PET resulted by the low physiological uptake in gastric wall could lead to improved imaging outcomes.

Regarding the immunohistochemical analysis of 17 primary gastric lesions specimens from 17 operated patients; Lin et al. reported that 76.5% of the gastric tumour demonstrated markedly positive FAP immunostaining; while 5.9% and 17.6% showed moderate and slight FAP immunostaining, respectively. Furthermore, SUVmax and TBR of Ga-FAPI were found to be moderately correlated with FAP expression [41].

**Fig. 1:**
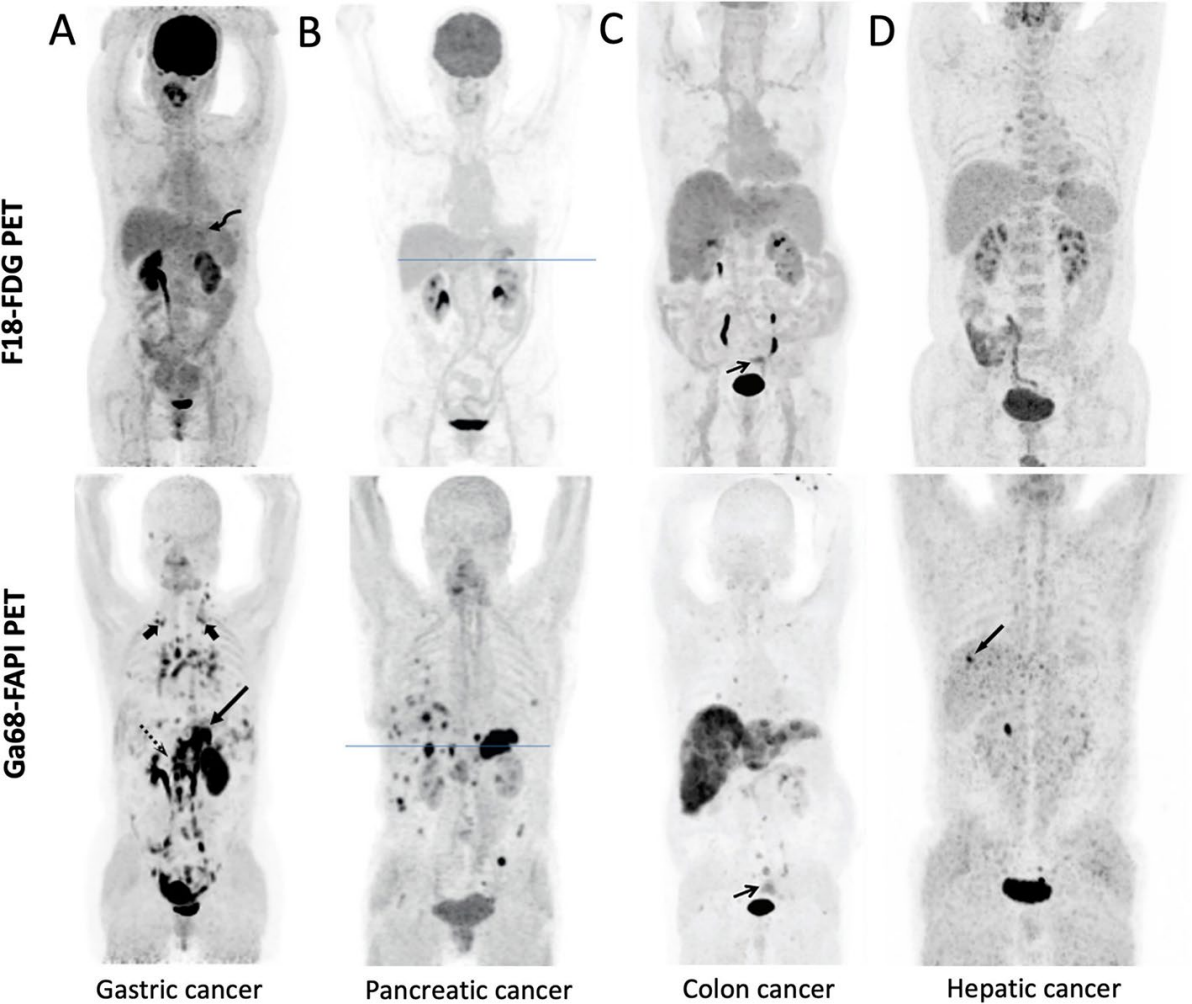
Superiority of FAPI PET over FDG PET in Gastrointestinal cancers. **A** Patient with poorly differentiated gastric adenocarcinoma underwent FAPI PET and FDG PET imaging. FAPI PET revealed high uptake in gastric cardia (slender arrow), paraaortic lymph nodes (dashed arrow) and supraclavicular lymph nodes (short arrows) which were negative on FDG PET. FDG PET revealed increased uptake in gastric anastomosis (bent arrow) which was negative on FAPI PET and eventually was confirmed as residual gastritis by biopsy. **B** Patient with pancreatic cancer underwent FAPI and FDG PET imaging. FAPI outperformed FDG PET in detecting primary tumours and metastatic lesions such as perihepatic lesions and pulmonary lesion which was confirmed as PDAC metastasis. **C** Patient with colon cancer underwent FAPI and FDG imaging for an initial assessment. The uptake in the primary tumour (black arrows) and metastatic lesions was higher on FAPI PET than that of FDG PET. **D** In a patient with moderately differentiated hepatocellular carcinoma, FAPI PET revealed a strongly FAPI-avid lesion in the right hepatic lobe with no positive findings on FDG PET. (Figures adapted from **A** Zhang et al. [43], **C** Pang et al. [42] and **D** Wang et al. [49], under a CC BY license. **B** was originally published in *JNM*. Röhrich M, Naumann P, Giesel FL, Choyke PL, Staudinger F, Wefers A, Liew DP, Kratochwil C, Rathke H, Liermann J, Herfarth K, Jäger D, Debus J, Haberkorn U, Lang M, Koerber SA. Impact of 68Ga-FAPI PET/CT Imaging on the Therapeutic Management of Primary and Recurrent Pancreatic Ductal Adenocarcinomas. J Nucl Med. 2021;62(6):779–786. © SNMMI [50])

In comparison with FDG PET, primary tumour, lymph node, and distant metastatic lesions were reported to have significantly higher tracer uptake on FAPI PET in most of the studies [41–46] (Fig. 1A), except for two studies reporting not significantly higher primary tumor SUVmax on FAPI PET [47, 48]. Moreover, Jiang et al. compared primary tumour SUVs in different tumour sizes and T stages. The mean SUVmax of FAPI in tumours greater than 4 cm was found to be higher than in tumours less than 4 cm (*P* = 0.0015). Similarly, the mean SUVmax of FAPI was found to be higher in T2-4 tumours than in T1 tumours (*P* = 0.0002) [48]. Consistent with those results, Lin et al., in their prospective study with 56 gastric cancer patients, reported that TBR on FAPI PET of T3-4, N1-3, and stage III-IV groups were significantly higher than that of T1-2, N0, and I-II groups; respectively [41].

**Fig. 2:**
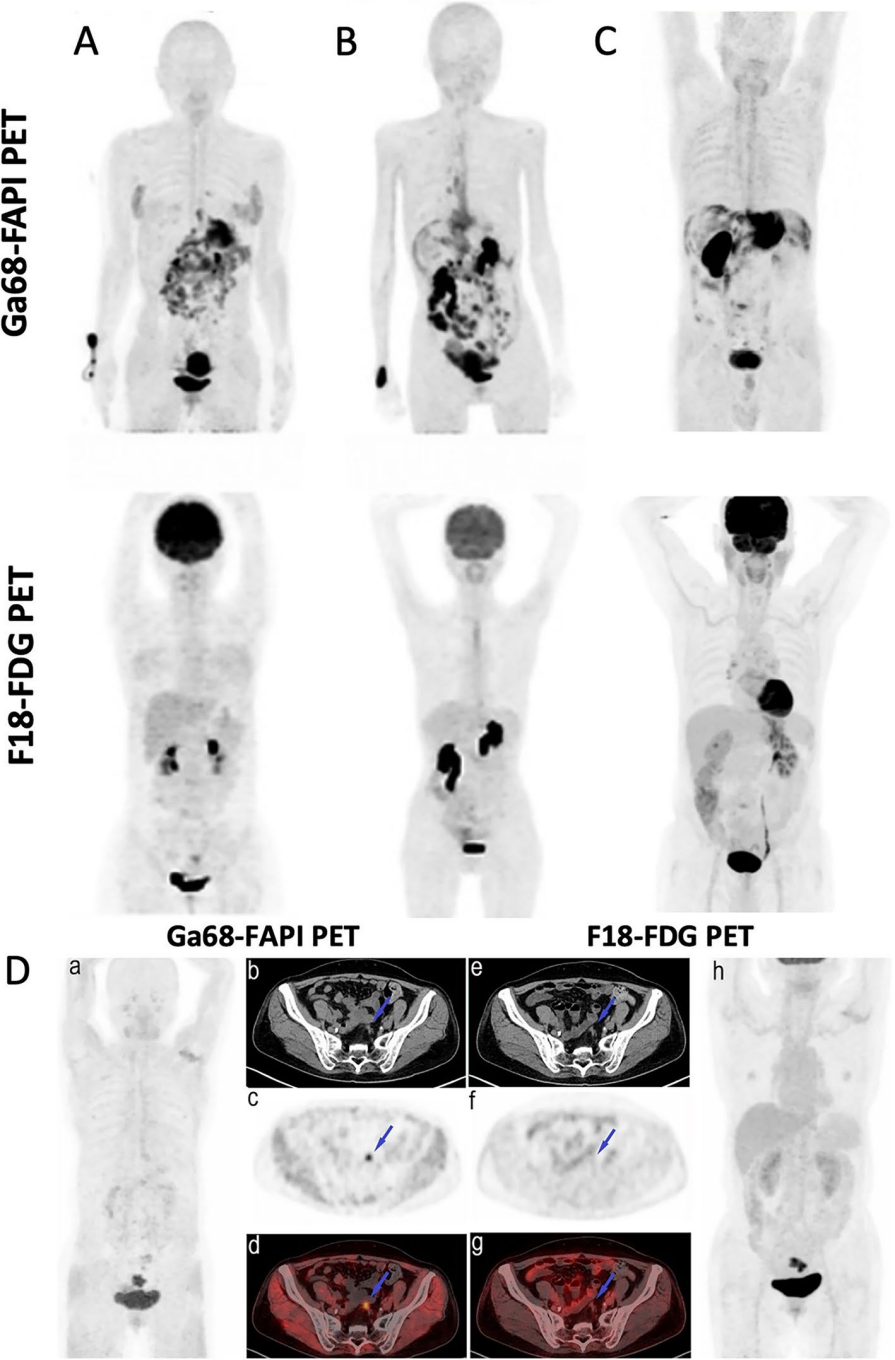
FAPI PET outperforming FDG PET in peritoneal lesion detection. **A** and **B** Patients with gastric cancer underwent FAPI PET and FDG PET for initial staging (**A**) and recurrence detection (**B**). FAPI PET images were superior to FDG PET in visualization of primary tumours and metastases, especially in peritoneal metastases in these images. **C** Patient with poorly differentiated gastric adenocarcinoma underwent FAPI PET and FDG PET/CT scans for staging. FAPI PET had higher uptake in primary tumour and peritoneal carcinomatosis compared to FDG PET. **D** Patient with rectal cancer underwent FAPI PET and FDG PET for staging (MIP images in a and h, respectively). Pelvic peritoneal carcinoma lesion was observed in FAPI PET images (blue arrows in b-d) due to intense tracer uptake. Conversely, low FDG uptake caused the small lesion to be difficult to detect (blue arrows in e–g). (Figure **D** adapted from Lin et al. [53], under a CC BY license. Figure **A** and **B** were originally published in *JNM*. Qin C, Shao F, Gai Y, Liu Q, Ruan W, Liu F, Hu F, Lan X. 68Ga-DOTA-FAPI-04 PET/MR in the Evaluation of Gastric Carcinomas: Comparison with 18F-FDG PET/CT. J Nucl Med. 2022 Jan;63(1):81–88. © SNMMI [45]. Figure **C** was adapted from the original figure with extraction of only FAPI PET and FDG PET MIP images from the bigger original figure which was published at reference [47] Kuten et al., under a CC BY license, link of the license: https://creativecommons.org/licenses/by/4.0/)

Considering the low sensitivity of FDG PET in gastric cancer; the sensitivity of FAPI PET was reported to be significantly higher in terms of the primary tumour, and distant metastatic lesion detection in almost all studies [42–44, 46, 51] (Table 1 and Supplementary Table 1- Additional file 1). Especially for peritoneal lesion detection, the superior sensitivity of FAPI PET was confirmed in all studies [41, 42, 45, 47, 51] (Fig. 2A-B). A meta-analysis including 5 studies, confirmed the higher sensitivity of FAPI PET over FDG PET in detection of primary tumour (100% vs. 84.43%), lymph node metastases (81.97% vs. 67.21%), and peritoneal metastases (100% vs. 44.74%) of gastric cancer [52].

Chen et al. conducted a multicentric, retrospective study including 34 patients with signet ring cell carcinoma, which is the gastric cancer type having the lowest sensitivity on FDG PET. FAPI PET revealed significantly higher uptake and detection performance than FDG PET in all lesion types including the primary tumour, local recurrence, lymph nodes, and metastatic lesions. Regarding the impact of FAPI PET on patient management, it was reported that TNM staging was altered in 14% of the patients during the initial evaluation. FAPI PET detected local recurrence in 42% and metastasis in 58% of the patients with suspected recurrence and negative FDG PET findings [44].

The diagnostic efficiency of FAPI PET and its potential impact on clinical management were investigated in an extensive prospective study including 120 patients, 69 of whom were gastric cancer patients. The diagnostic accuracy of FAPI PET was found to be much higher than that of conventional imaging (CI) and FDG PET (94.3% vs. 66.1% and 63.0%, respectively, both *p* < 0.001, number of the scans: 70 FAPI PET, 62 CI and 27 FDG PET) in gastric cancer patients both in the initial staging and restaging group. In addition, the accordance rate of FAPI PET-guided treatment in comparison with the reference standard was also reported to be much higher than that of CI and FDG PET (97.1% vs. 75.8% and 70.4%, respectively, both *p* < 0.001) in gastric cancer patients [54].

Furthermore, Kuten et al. and Lin et al. investigated the performance of FAPI PET for chemotherapy response evaluation in gastric cancer in a very limited number of patients. Lin et al. reported decrease in malign lesions’ uptake on follow-up FAPI PET in two patients indicating the response to chemotherapy, Kuten et al. declared the same in one patient and progression in another patient with new lesions and increased uptake. They both reported that FAPI PET has great potential to monitor therapy response [41, 47].

Regarding the therapy response in gastric cancer, there is an interesting study conducted with a different perspective to evaluate Ga-FAPI PET in the assessment of immunosuppressive tumour microenvironment (TME) in gastric cancer [55]. Since CAFs have significant roles in cancer tissue such as involving in the immune system modulation, FAP-expressing CAFs were recently associated with immunosuppression and resistance to immunotherapies, confirmed in preclinical studies [56]. However, it still remains unclear whether this CAF-mediated immunosuppressive function is relevant in human tumours and if so, what are the mechanisms involved [57]. To investigate further, Rong et al. correlated FAPI PET imaging findings with responses to immune checkpoint blockade therapy in gastric cancer patients. Eventually, they concluded that FAPI PET may noninvasively estimate the immunosuppressive TME and serve as a predictive biomarker of survival and anti-tumour immune response who received immune checkpoint blockade therapies [55].

**Fig. 3:**
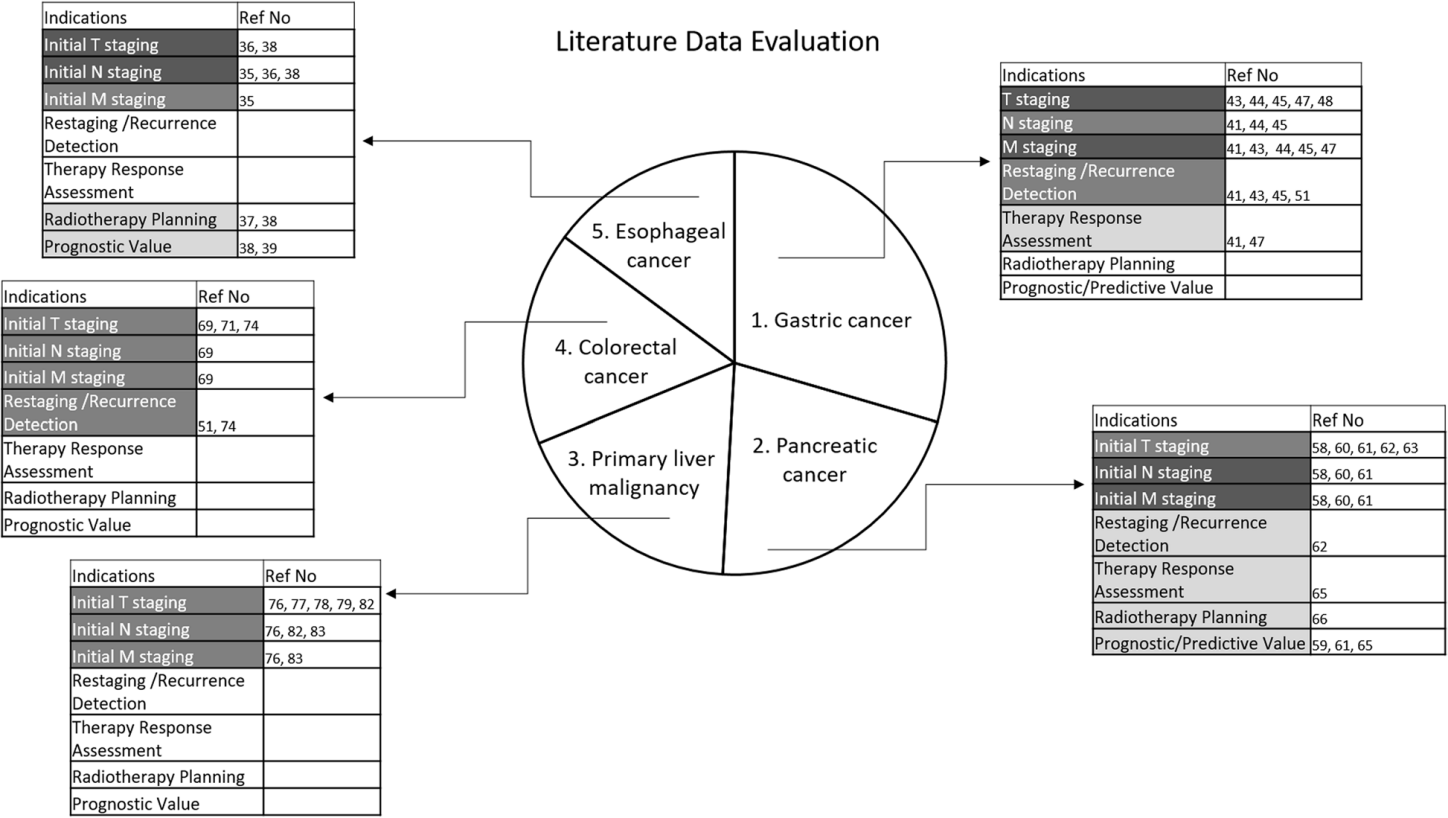
Literature Data Evaluation in Gastrointestinal Malignancies. Pie chart percentages represent the number of included patients with each cancer type in the evaluated studies. The highest number of patients in the studies belongs to gastric cancer, while the lowest number belongs to esophageal cancer. The gray colour-code tables in each cancer type illustrate the possible FAPI PET indication, based on the interpretation of the available literature data. The darkest gray indicates highly promising literature data in this indication. The lighter gray indicates there is promising data in this indication, however there are also some contradictory reports (e.g. low specificity). The lightest gray suggests encouraging data in this indication but further exploration is still needed. The white colour indicates that there is not enough data for interpretation. The corresponding reference articles are provided in the tables

In conclusion, for the gastric cancer patient group in which FDG PET shows low performance, FAPI PET has great potential regarding the higher uptake and superior detectability for gastric cancer lesions, even better with bigger tumours, higher stages, and signet ring cell carcinoma histological type. Moreover, there are encouraging preliminary results regarding therapy response assessment on FAPI PET, to be investigated further. More comprehensive studies are required to concretize the role of FAPI PET in gastric cancer management (Fig. 3).

### Pancreatic cancer

FDG PET/CT has relatively high sensitivity and specificity in pancreatic adenocarcinoma diagnosis and staging. However, the technique is hampered by potential false positive findings due to FDG-avid non-malignant lesions (e.g., chronic pancreatitis), as well as false negativity in 10% of pancreatic cancer patients [58, 59].

Pancreatic ductal adenocarcinoma (PDAC) is characterized by an intense stromal desmoplastic reaction surrounding cancer cells, and CAFs are the main effector cells in the desmoplastic tissue [60]. Thus, pancreatic cancer is expected to show intense FAP expression. Consistent with this expectation, FAP IHC scoring demonstrated strong FAP expression in 50–100% of pancreatic cancer cases [32]. Correlated with the high FAP expression in tissue, high uptake on FAPI PET has been demonstrated in pancreatic cancer [61, 62].

In their prospective study with 33 patients, Zhang et al. reported that pancreatic cancer primary tumour SUVmax and TBR (normalised SUVmax = tumour SUVmax/average background SUV) were significantly higher on FAPI PET compared to FDG PET [63]. Similar findings were reported by Pang et al. and Liu et al., declaring that FAPI surpassed FDG in terms of both SUV and sensitivity for primary tumour and lymph node metastases of pancreatic cancer [61, 64] (Table 1 and Supplementary Table 1- Additional file 1) (Fig. 1B), even though not statistically significant for lymph nodes detection sensitivity in the cohort of Pang et al. On the other hand, Pang et al. found FDG PET had higher specificity than FAPI PET for primary tumours (60% vs. 30%, *p* = 0.25), they reported similar specificity percentages between them for distant metastastic sites (FDG PET 73.9% vs. FAPI PET 65.2%, *p* = 0.5) and higher specificity of FAPI PET than FDG PET for lymph node metastases (85.7% vs. 81%, *p* = 0.065) [61] while Liu et al. declared similar specificities for all lesion types [64] (Table 1 and Supplementary Table 1- Additional file 1). The low specificity of FAPI PET in primary tumour detection can be attributed to radiolabeled FAPI uptake in non-oncological conditions like pancreatitis and Ig4-related disease. In that matter, Pang et al. demonstrated that dual time point (3-h delayed) imaging could differentiate pancreatic cancer from pancreatitis [61], supporting the similar results of Röhrich et al. [50] and Glatting et al. [65]. Therefore, it seems that dual time point imaging can improve the specificity of FAPI PET in primary pancreatic tumour lesions.

In contrast to Zhang et al. reporting higher SUVmax and normalised SUVmax for pancreatic cancer liver metastasis on FDG PET (5 patients, mean SUVmax values 6.0 vs. 8.6; normalised SUVmax 3.5 vs. 6.4 [63]), Pang et al. and Sahin et al. declared the superiority of FAPI PET over FDG PET in liver metastasis [61, 66] (12 patients, median SUVmax 7.4 vs. 3.7 [61]; 9 patients, median SUVmax 5.2 vs. 4.6, median TBR 4.1 vs. 1.2 [66]). In addition, Liu et al. reported absence of significant difference in liver metastases SUVmax (7.1 ± 2.4 vs. 6.6 ± 2.4, p > 0.05) and significantly higher TBR on FAPI PET (5.7 ± 3.2 vs. 3.2 ± 1.3, *p* < 0.0001) including 111 lesions on FAPI PET and 79 lesions on FDG PET [64].

In a retrospective study including a cohort of 37 patients, Ding et al. demonstrated the prognostic value of FAPI PET in resectable PDAC. They reported SUVmax on FAPI PET as an independent negative prognostic factor for recurrence-free survival and TPF (total pancreatic FAP expression) on FAPI PET for overall survival [62]. Zhu et al., in a recent prospective study, analyzed baseline FAPI PET variables in 37 inoperable PDAC patients and MTV was found to be an independent predictor of OS [67]. In a study of 19 PDAC patients, Röhrich et al. investigated the impact of FAPI PET on therapeutic management compared to standard-of-care imaging by CeCT (contrast-enhanced CT). They reported that FAPI PET led to upstaging in 8 of 12 recurrent patients, downstaging in 1 of 12 recurrent patients, and upstaging in 1 of 7 patients with the primary disease [50]. In the same line, in their 36 patients cohort, Pang et al. also found FAPI PET superior to FDG PET and CeCT in terms of TNM staging [61].

Zhu et al. explored whether changes in FAPI PET variables before and after one cycle of chemotherapy in 17 inoperable PDAC patients could predict therapy response and survival. They declared greater changes in SUVmax, MTV and TLF (total lesion FAP expression) in good responders than in the poor responders. Moreover, they determined cutoff values for the changes in MTV (> -4.95) and TLF (> -77.83) which may be clinically meaningful for identifying patients at high risk of disease progression [67].

Liermann et al. declared FAPI PET enables GTV contouring in locally recurrent pancreatic cancer patients and demonstrated good results compared to manually contoured target volumes in seven patients. They concluded FAPI PET can be used as an additional imaging modality to improve decision-making in target definition, especially in inconclusive cases [68].

To conclude, FAPI PET has been shown to be superior in terms of uptake and detectability of pancreatic cancer primary tumour and metastatic lesions compared to FDG PET and CeCT. Additionally, it has a potential impact on therapeutic management both for primary and recurrent disease and also a potential prognostic/predictive value in PDAC. Furthermore, dual-time imaging may aid in increasing the specificity for pancreatic malignancy detection. Further well controlled prospective studies, preferrably with multiple time point FAPI PET imaging are needed before introducing this technique in routine clinical practice (Fig. 3).

### Colorectal cancer

FDG PET/CT is a valuable tool in colorectal cancer recurrence detection and therapy response assessment. However, it is not useful for initial staging due to a low sensitivity for detection of locoregional lymph node involvement, small liver lesions and peritoneal metastasis [69, 70].

FAP IHC by cancer type performed in the tissue microarrays demonstrated strong FAP expression in 50–100% of colorectal cancer cases [32]. In the same study, the positive correlation between FAPI PET SUV values and FAP IHC score expression was also demonstrated [32]. Therefore, most of the colorectal cancer lesions are expected to have high uptake on FAPI PET. Indeed, studies demonstrated the relatively high uptake in the primary colorectal tumoural lesions with mean SUVmax values above 10 [42, 71, 72].

Pang et al. and Chen et al. reported higher SUVmax values of primary colorectal tumour lesions on FAPI PET compared to FDG PET (median SUVmax: 15.9 vs. 7.9; median SUVmax 12.22 vs. 8.29, respectively) [42, 72] (Fig. 1C). On the other hand, in the more extensive prospective study with 39 patients, Komek et al. declared that primary tumour SUV values on FDG PET were significantly higher than on FAPI PET (mean SUVmax: 18.93 vs. 11.54, *p* < 0.001) [71]; similar findings with Lin et al. in 61 patients (mean SUVmax 11.4 vs. 9.7, *p* = 0.09) [53]. However, in terms of the primary tumourTBR, they both showed the opposite, meaning higher TBR on FAPI PET than on FDG PET [53, 71]. Additionally, the primary tumour detection sensitivity of FAPI PET was reported to be equal to FDG PET: 100% on both [53, 71] (Supplementary Table 1- Additional file 1).

In the same prospective study of Komek et al., the comparison of lymph node invasion and distant metastatic lesions (bone, visceral, peritoneal, and lung metastases) revealed higher SUV, TBR values and sensitivity of FAPI PET compared to FDG PET (Table 1 and Supplementary Table 1- Additional file 1), except for liver metastasis where SUVmax and TBR values (detected in 7 patients) were significantly higher on FDG PET compared to FAPI PET [71]. The latter finding was not in alignment with the majority of the studies including other cancer types [42, 72–74] nor with the theoretical expectations given the very low uptake of FAPI in the liver. Sahin et al., in their study including the colorectal cancer subgroup of 15 patients with liver metastasis, reported that liver metastasis SUVmax, TBR values and detection sensitivity were higher on FAPI PET than that on FDG PET even though there was no statistically significant difference in SUVs [66]. Lin et al. also reported the non-significant difference between SUVmax values of liver metastases in 9 patients and the significant superiority of TBR on FAPI PET over FDG PET [53]. Higher liver metastasis TBR values, combined with non-superiority of the SUVs in these studies, confirms the significance of low liver parenchymal uptake on FAPI PET lesion detection (Fig. 1C).

Koerber et al., in their study including 15 colorectal cancer patients and 7 anal cancer patients, investigated the change in tumour staging and oncologic management after FAPI PET compared with standard imaging. They concluded that in treatment-naive patients, TNM was changed in 50%, whereas in patients with metastases, new findings occurred in 47%. In total, FAPI imaging was reported to cause a high, medium, and low change in oncologic management in 19%, 33%, and 29% of the patients, respectively [75]. According to Lin et al., in a cohort of 61 colorectal cancer patients, FAPI PET-based TNM staging resulted in up-staging in 16% and down-staging in 8% of the patients compared to FDG PET, along with treatment changes in 21% of the patients [53].

In another extensive prospective study investigating the diagnostic efficiency of FAPI PET and its potential impact on clinical management, including 21 colon and 17 rectal cancer patients, Qin et al. reported that the diagnostic accuracy of FAPI PET was higher than that of CI and FDG PET (FAPI PET, CI, FDG PET diagnostic accuracy: 97.4%, 63.9% and 90.9%, resp.) in colorectal cancer patients although not significantly between FAPI PET and FDG PET (diagnostic accuracy comparison between imaging modalities FAPI vs. CI *p* < 0.001; FAPI vs. FDG *p* = 0.402). In addition, the accordance rate of treatment management implications prompted by FAPI PET in comparison with the reference standard was also found to be higher than that of CI and FDG PET (97.4% vs. 72.2% and 90.9%, respectively, FAPI vs. CI *p* < 0.001; FAPI vs. FDG *p* = 0.402) although not significantly between FAPI PET and FDG PET [54].

Given the relatively good performance of FDG PET on colorectal cancer; FAPI PET has been shown not only to have similar performance in the detectability and uptake of primary colorectal lesions but also to be superior to FDG for lymph node and metastatic lesions. In addition, when compared to standard CI and FDG PET, FAPI PET demonstrated a benefit in patient management in both initial staging and restaging. Finally, the role of FAPI PET in colorectal cancer response assessment is still not explored (Fig. 3).

### Hepatic malignancies and cirrhosis

#### Hepatocellular carcinoma (HCC)

The sensitivity of FDG PET/CT for HCC, representing the most frequent primary liver cancer, is about 50% or even lower due to enzyme (glucose transporter-1 and glucose-6-phosphatase) expression variations [76]. The lower sensitivity of FDG is related to the lack of retention (i.e. high wash out) of the FDG in well differentiated tumours.

Despite HCC being reported to exhibit a relatively low FAP expression [32] and an intermediate level of mean SUVmax (6–12) compared to other cancer types [33], FAPI PET has demonstrated promising outcomes for HCC primarily because of its minimal liver uptake, resulting in very low background activity.

In HCC, most studies comparing FAPI PET with FDG PET, reported higher SUVmax (9.7 vs. 5.5 [77], 8.5 vs. 4.9 [78], 11.5 vs. 4.3 [79]) and TBR (TBR 7.9 vs. 2.0 [77], TBR 7.1 vs. 2.4 [78], TBR 5.0 vs. 1.2 [79]) [77–79] (Fig. 1D). Even though Wang et al. declared comparable SUVs on FAPI and FDG PET (mean SUVmax 6.96 vs. 5.89, respectively), they confirmed the higher TBR on FAPI PET (TBR 11.9 vs. 3.14) in agreement with other studies [49].

The lesion-based sensitivity of HCC was found to be significantly higher in tumours smaller than 2 cm diameter (FAPI PET 69% vs. FDG PET 19%, *p* = 0.008) and grade I-II tumours (FAPI PET 83% vs. FDG PET 33%, *p* = 0.031). FAPI PET sensitivity in larger and higher grade HCC lesions was also higher than that of FDG PET, although not statistically significant [49].

Guo et al. observed a positive correlation between the pathological grade and FAPI PET uptake of HCC lesions. They found a significant difference between uptake of the poorly, moderately and well-differentiated HCC lesions [79]. The lower uptake in well-differentiated and the higher uptake in poorly-differentiated lesions were also consistent with the results of Shi et al. [80].

#### Cholangiocellular carcinoma (CCC)

Comparing the uptake between different types of primary hepatic malignancies on FAPI PET, Shi et al. and Siripongsatian et al. declared a significantly higher uptake of CCC compared to HCC [77, 80]. These findings are consistent with the hallmark histopathological feature of CCC which is the dense desmoplastic stroma including the deposition of CAFs and connective tissue [81]. The higher FAP expression in CCC was also demonstrated in immunohistochemical staining of the tumour tissues [78, 80].

In all studies comparing the FAPI PET and FDG PET in CCC lesions, significantly higher primary tumour SUVmax (SUVmax 19.8 vs. 4.9 [77], SUVmax 14.1 vs. 9.2 [78], SUVmax 16.5 vs. 4.2 [79], SUVmax 19 vs. 11.9 [82], SUVmax 14.5 vs. 5.2 [83]) and TBR values (TBR 21.1 vs. 1.5 [77], TBR 10.9 vs. 2.5 [78], TBR 7.0 vs. 1.5 [79], TBR 20.6 vs. 4.6 [82], TBR(blood) 9.7 vs. 2.4 and TBR(liver) 12.1 vs. 1.9 [83]) on FAPI PET were declared [77–79, 83].

In a recent study including 6 intrahepatic and 4 extrahepatic CCC patients, FAPI PET sensitivity surpassed FDG PET in detecting lymph node and distant metastatic lesions [83]. Similar findings were reported by Jinghua et al. in primary tumour and metastases detection on FAPI PET in a cohort of intra/extrahepatic CCC and gall bladder carcinoma [82] (Supplementary Table 1- Additional file 1).

#### Primary hepatic tumours (HCC and CCC)

All studies on this topic agreed on the higher sensitivity and higher TBR of primary hepatic malignancies (HCC and CCC) on FAPI PET compared to FDG PET [49, 77–79] (Table 1 and Supplementary Table 1- Additional file 1). SUV values of the hepatic lesions are not higher than on FDG PET, however, due to the very low uptake in normal liver tissue, FAPI PET has the superiority in terms of TBR and sensitivity.

Shi et al.’s study is the only study reporting on the FAPI PET specificity for detecting hepatic lesions (HCC and CCC). They declared 100% specificity both on FAPI and FDG PET [78] (Supplementary Table 1- Additional file 1). However, false positivities due to post-therapy inflammatory changes or other benign liver lesions (angiomyolipoma, hepatic granuloma, focal nodular hyperplasia) were detected in other studies [49, 77, 79]. Post-therapy inflammatory changes were presented as either focal [77] or a diffuse pattern. Hence, post-therapy changes and inflammatory lesions in the liver may cause false positive results on FAPI PET.

Furthermore, another prospective study investigating FAPI PET using a F18-labeled FAPI tracer in the evaluation of non-FDG avid liver lesions demonstrated the high sensitivity of FAPI PET in detecting liver malignancies (sensitivity of 97% for HCC lesions and 100% for non-HCC malignancies) [84]. That means that FAPI PET is a promising option for the characterization of non-FDG avid liver lesions detected on CT or MRI. However, they also showed that inflammatory benign liver lesions such as inflammatory pseudotumours (with or without IgG4-related disease), cholangitis, and hepatic granulomas have intense FAPI uptake [84]. These false positivity may hamper the specificity of FAPI PET in liver malignancy detection.

Regarding the metastatic lesions of primary hepatic tumours (HCC and CCC), Siripongsatian et al. reported that regional lymph node metastases had higher median SUV values (8.35 vs. 4.61), TBR (4.51 vs. 2.28), and higher sensitivity (100% vs. 58%) on FAPI PET compared to FDG PET. They also declared the same results for distant metastatic lesions, except for the sensitivity which they found to be comparable with FDG PET (96% vs. 89%) even though the number of lesions on FAPI PET was higher [77] (Table 1 and Supplementary Table 1- Additional file 1). Guo et al. also reported higher SUV values of distant metastatic lesions (peritoneal lesions median SUVmax 7.1 vs. 2.62, bone metastasis median SUVmax 6.72 vs. 2.83) except lung metastasis whose median SUVmax values were found to be similar (1.58 vs. 1.42) [79].

#### Cirrhosis

Cirrhosis is featured by increased intrahepatic FAP expression, which was linked to the severity of liver fibrosis [85]. Therefore, it is expected to have higher uptake in the cirrhotic liver than in the non-cirrhotic liver on FAPI PET. This was confirmed in several studies [49, 79, 80] although no significant difference was also reported by Shi et al. [78]. Additionally, Wang et al. and Guo et al. emphasized the absence of significant difference between cirrhotic and non-cirrhotic liver uptake on FDG PET [49, 79]. The significant difference in cirrhotic liver uptake means that FAPI PET can be used to detect and quantify cirrhotic activity. Guo et al. declared a significantly higher TBR of the intrahepatic lesions which were present in the non-cirrhotic liver than in the lesions present in the cirrhotic liver; meaning that the detectability of the intrahepatic lesions in the cirrhotic liver on FAPI PET might be lower than the lesions present in the non-cirrhotic liver due to masking of small lesions on the cirrhotic liver [79].

To sum up, FAPI PET has great potential to overcome the limitation of FDG PET in detecting primary liver malignancies, especially in lower grade and smaller tumours. However, whether the specificity of FAPI PET surpasses that of FDG PET should be further investigated in both initial diagnosis and post-therapy evaluation (Fig. 3). On the other hand, FAPI PET also has a promising potential to detect cirrhotic activity.

### Peritoneal carcinomatosis

The variable FDG avidity among different primary tumour types may be the cause of the controversial diagnostic performance of FDG PET/CT in peritoneal carcinomatosis [86, 87]. Moreover, the physiological accumulation of FDG in the intestinal tract makes it difficult to obtain clear images due to low tumour-to-background contrast in this area [88]. In contrast, studies have shown the absence of physiological accumulation of FAPI tracer in the intestinal tract which should facilitate peritoneal lesion detection [33, 72, 73].

Peritoneal carcinomatosis often involves low-volume lesions. Since the tumour stroma volume can be bigger than the tumour cells’ volume, stroma-targeted PET imaging can be more sensitive than FDG PET for small peritoneal lesions with sufficient FAP-expressing stroma [89]. Thus, this is another reason why FAP-targeted imaging is a great option for peritoneal carcinomatosis imaging.

Zhao et al. investigated the role of FAPI PET compared to FDG PET to evaluate peritoneal carcinomatosis in various types of cancer. In their cohort with 46 patients, they reported significantly higher SUV values (median SUV: 9.8 vs. 3.5; *P* < 0.001), PCI (peritoneal cancer index) scores (18 vs.6; *P* < 0.001), and sensitivity (98% vs. 72%; *P* = 0.002) with FAPI PET compared to FDG PET [88]. Furthermore, Elboga et al., in their study regarding gastrointestinal malignancies with peritoneal involvement, also reported that peritoneal lesion SUVmax values were significantly higher on FAPI PET than on FDG PET [74]. Zhao et al. emphasized the superiority of FAPI PET particularly in gastric cancer patients [88]. Additionally, in their study with gastric cancer patients, Kuten et al. reported the sensitivity of FAPI PET as 100% and FDG PET as 0% for peritoneal carcinomatosis detection [47]. These findings demonstrated the superiority of FAPI PET in peritoneal carcinomatosis especially in gastric cancer (Fig. 2A-D).

In terms of PCI score; Zhao et al. reported that the number of patients with PCI > 20 based on FAPI PET was markedly higher than that based on FDG PET [88]. This finding is also significant because the cut-off value of 20 has a major impact on patient management. They also examined the different patterns of peritoneal carcinomatosis (omental-cake-type pattern and nodular-type pattern). The sensitivity difference between FAPI PET and FDG PET is even higher regarding nodular-type lesion detection; 92.74% vs. 39.52%, *P* < 0.001. This can be attributed to FDG-negativity especially in low volume lesions; the median size of FDG-negative peritoneal implants was reported to be 1.01 cm [88].

Briefly, FAPI PET is probably a better imaging modality than FDG PET for sufficiently accurate preoperative assessment of peritoneal carcinomatosis. Further prospective studies are required to compare its accuracy with dedicated MRI and diagnostic laparoscopy as the reference diagnosis.

## Conclusion

The current research status of FAP-targeted imaging in gastrointestinal tumours is described in this review. Based on current clinical studies, FAPI PET imaging is very promising for applications in various gastrointestinal cancers. It outperforms FDG PET in many aspects, particularly in the detection of the primary tumour and metastatic lesions and better tumour delineation. Besides the superiority for tumoural detection, FAPI PET imaging also has advantages over FDG PET in patient preparation such as no requirement for fasting and early imaging. However, the available evidence has shown that FAPI is not an entirely tumour-specific agent, possibly due to a fibrotic reaction and FAP activation in chronic inflammation, increased radiolabeled FAPI uptake has been demonstrated in non-malignant conditions in recent studies. Notably, FAPI cannot be considered more tumour-specific than FDG, nuclear medicine physicians must be aware of the potential pitfalls and consider them while interpreting. On the other hand, this phenomenon opens indications for FAPI PET in non-oncological conditions such as liver cirrhosis. Eventually, well-designed and more extensive clinical trials are required to explore the FAP-targeted diagnostic applications and clarify its role in each clinical setting.

### Supplementary Information

**Additional file 1: Supplementary Table 1.** Sensitivity and specificity values of FAPI PET and FDG PET in gastrointestinal cancer types. Supplementary Table 1 demonstrates the sensitivity, specificity and p values of the comparison between FAPI PET and FDG PET imaging in each considered cancer type including the detection of primary tumour and metastatic lesions. “NA”: not applicable, PM: peritoneal metastases, LM: liver metastases, * Detected lesion numbers on PET scans without fusion with anatomical imaging modalities were provided. ** Detected abdominal lymph nodes on PET scans were provided.

## Data Availability

Not applicable.

## Abbreviations

CAF: Cancer associated fibroblasts
CC: Creative commons
CCC: Cholangiocellular carcinoma
CeCT: Contrast enhanced computerized tomography
CI: Conventional imaging
CT: Computerized tomography
FAP: Fibroblast activation protein
FAPI: Fibroblast activation protein inhibitor
FDG: Fluorodeoxyglucose
GTV: Gross tumour volume
HCC: Hepatocellular carcinoma
IHC: Immunohistochemistry
MIP: Maximum intensity projection
MRI: Magnetic resonance imaging
MTV: Metabolic tumour volume
PCI: Peritoneal cancer index
PDAC: Pancreatic ductal adenocarcinoma
PET: Positron emission tomography
PET/CT: Positron emission tomography/ computerized tomography
SUV: Standardized uptake volume
TBR: Tumour/background ratio
TLF: Total lesion FAP expression
TL-FAPI: Total lesion-FAPI
TME: Tumour microenvironment
TNM: Tumour-node-metastasis
